# BioSemAF-BiLSTM: a protein sequence feature extraction framework based on semantic and evolutionary information

**DOI:** 10.3389/fgene.2025.1616880

**Published:** 2025-09-17

**Authors:** Zihan Zhang, Yixuan Wang

**Affiliations:** ^1^ Haide College, Ocean University of China, Qingdao, China; ^2^ School of Medicine, Nankai University, Tianjin, China

**Keywords:** adaptive fusion, bidirectional LSTM neural network, bioinformation encoding, information loss, Kolmogorov complexity, post-translational modification, protein sequence embedding, sulfenylation site

## Abstract

S-sulfenylation is a critical post-translational modification that plays an important role in regulating protein function, redox signaling, and maintaining cellular homeostasis. Accurate identification of S-sulfenylation sites is essential for understanding its biological significance and relevance to disease. However, the exclusive detection of S-sulfenylation sites through experimental methods remains challenging, as these approaches are often time-consuming and costly. Motivated by this issue, the present work proposed a deep learning-based computational framework, named BioSemAF-BiLSTM, which integrated evolutionary and semantic features to improve the prediction performance of S-sulfenylation sites. The framework employed fastText to generate subword-based sequence embeddings that captured local contextual information, and employed position-specific scoring matrices (PSSMs) to extract evolutionary conservation features. Importantly, we also quantitatively evaluated feature sufficiency at the protein sequence level using a sequence compression-based measure approximating Kolmogorov complexity, revealing an 11% information loss rate in predictive modeling using these features. These representations were subsequently fed into a bidirectional long short-term memory (BiLSTM) network to model long-range dependencies, and were further refined via an adaptive feature fusion module to enhance feature interaction. Experimental results on a benchmark dataset demonstrated that the model significantly outperformed conventional machine learning methods and current state-of-the-art deep learning approaches, achieving an accuracy of 89.32% on an independent test. It demonstrated improved sensitivity and specificity, effectively bridging the gap between bioinformatics and deep learning, and offered a robust computational tool for predicting post-translational modification sites.

## 1 Introduction

Post-translational modifications (PTMs) serve as key mechanisms for regulating protein function, stability, and interactions (Mann and Jensen, 2003). Recognized as central processes in biological activities, PTMs encompass diverse types, including phosphorylation, acetylation, methylation, and redox modifications. Among these, redox-related modifications have attracted considerable attention, particularly those involving cysteine residues. The thiol group (-SH) of cysteine residues is highly sensitive to reactive oxygen species (ROS), undergoing a variety of oxidative modifications that are crucial for signal transduction and cellular stress responses (Qian et al., 2024; Walsh and Jefferis, 2006).

Among cysteine modifications, S-sulfenylation is distinguished by its dynamic and reversible nature, which endows it with unique biological significance. S-sulfenylation entails the reaction of thiol groups with reactive oxygen species (ROS), such as hydrogen peroxide, leading to the formation of -SOH. This modification modulates protein activity, maintains cellular redox homeostasis, and plays a key role in signal transduction (Paulsen and Carroll, 2013). S-sulfenylation not only protects cysteine residues from irreversible oxidation but also plays critical roles in various pathological conditions, such as cancer, neurodegenerative disorders, and cardiovascular diseases (Anjo et al., 2024; Chahla et al., 2024). Therefore, precise identification and localization of S-sulfenylation sites are essential for elucidating their biological functions and understanding disease-associated mechanisms. This necessity forms the basis for ongoing efforts to develop computational approaches for S-sulfenylation site prediction.

Traditionally, the prediction of S-sulfenylation sites has relied on biological experiments that detect specific cysteine residues undergoing modification through direct or indirect approaches. Early studies predominantly used radioactive isotope-based labeling techniques, in which hydrogen peroxide reacts with cysteine to form thiol intermediates detectable using isotope-labeled chemical probes (Leonard et al., 2009; Yang et al., 2015). However, these methods suffered from low sensitivity and often altered protein structures, thereby restricting their broader applicability. With technological advancements, chemical probe-based labeling strategies have emerged as mainstream tools in S-sulfenylation research (Paulsen and Carroll, 2013). For example, in the early 2000s, biochemists developed disulfide-based probes that selectively bind to S-sulfenylation sites, enabling precise localization via mass spectrometry (MS) analysis (Leonard et al., 2009; Yang et al., 2015; Yang et al., 2014). While these approaches significantly improved sensitivity, challenges associated with sample complexity and quantitative analysis still remained. During the 2010s, advanced quantitative proteomics techniques were incorporated into S-sulfenylation research. For instance, Paulsen et al. (Paulsen et al., 2011) developed the DYn-2 probe, which selectively captures -SOH modification sites and, when combined with high-resolution MS, facilitates large-scale identification of such modifications. In addition, click chemistry-based biotinylated probes were widely adopted, providing powerful tools for high-throughput detection of modified cysteine residues (Leonard et al., 2009; Yang et al., 2015). These traditional biochemical methods have established a strong foundation for S-sulfenylation site research and elucidated the dynamic regulation of modifications under various physiological conditions.

Traditional machine learning approaches have long played a central role in the early development of computational tools for S-sulfenylation site prediction. These methods typically fall under the broader category of protein sequence site prediction–an important area of bioinformatics that aims to identify functional residues. Accurate sequence representations are also critical for a variety of related prediction tasks in bioinformatics, such as determining protein secondary structures (Jones, 1999). In this study, however, we focus on PTM site prediction, where a number of traditional machine learning approaches have been developed. For instance, Chou introduced the concept of pseudo-amino acid composition (PseAAC) to encode protein sequence features, and Qiu et al. combined it with random forest (RF) for phosphorylation site prediction (Chou, 2001; Qiu et al., 2017). Subsequently, Caragea et al. (2007) successfully predicted glycosylation sites using a support vector machine (SVM). Bui et al. (2015) developed MDD-SOH, one of the earliest and most foundational models in this field. MDD-SOH utilizes sequence composition and physicochemical properties of proteins as features, employing an SVM-based maximum dependency decomposition (MDD) for classification. Similarly, Xu et al. proposed the iSulf-Cys model, which utilized physicochemical and distribution properties of amino acids and employed a support vector machine for classification (Xu et al., 2016). Ju and Wang. (2018) introduced the Sulf_FSVM model, combining mRMR (maximum relevance minimum redundancy) features and employing a fuzzy support vector machine as the classifier.

Although machine learning approaches have achieved some success, as discussed above, they have relied heavily on complex feature engineering and prior knowledge, with limited adaptability to large-scale datasets, paving the way for deep learning approaches. With the rapid advancement of deep learning, researchers have recognized its superiority in processing sequential data. Deep learning effectively captures intricate patterns within raw data and uncovers latent dependencies in high-dimensional spaces. Consequently, deep learning has been widely adopted for protein sequence analysis and site prediction. Foundational advances in deep learning (LeCun et al., 2015) and comprehensive reviews on its applications to molecular and protein modeling (Li et al., 2022; Qin et al., 2024; Boadu et al., 2024) have catalyzed this trend. In recent years, several deep learning-based models have been developed specifically for the prediction of S-sulfenylation sites, each adopting distinct strategies for sequence representation and feature extraction.

Khan and Pi. (2020) developed nSSPred, which combines nSegmented optimization with a joint feature encoder and applies 2D convolutional neural networks (2D-CNNs) to predict S-sulfenylation sites. Do et al. (2020) proposed fastSulf-DNN, extracting bio-subword-level features and employing natural language processing techniques to encode protein sequences for deep neural network (DNN) classification. Ning and Li (2022) presented DLF-Sul, a multi-module framework that integrates binary encoding, BLOSUM62, and amino acid indices, followed by BiLSTM for sequence modeling, and further applies multi-head self-attention and CNNs for feature refinement before classification via fully connected layers. However, these methods share key limitations (Do et al., 2020): 1. A strong dependence on expert-crafted features restricts the comprehensive capture of biological information; 2. Limited capacity for semantic understanding hampers accurate identification of amino acid functions and interdependencies; 3. Weak adaptability to heterogeneous features constrains dynamic, task-aware feature weighting; 4. A focus on local patterns undermines the modeling of long-range dependencies in sequences. Together, these issues limit the predictive performance of existing approaches for S-sulfenylation sites.

Beyond architectural innovations, recent studies have also highlighted the importance of evaluating how much biological information is preserved in a given sequence representation, for example, through compression- and complexity-based measures (Orlov and Orlov, 2023). Inspired by these insights, this work further incorporates a Kolmogorov-complexity–inspired, compression-based evaluation to quantify feature sufficiency and to guide resampling strategy selection, complementing the predictive modeling rather than replacing it.

We systematically evaluated existing computational tools for S-sulfenylation site prediction. To address identified limitations, a novel predictive framework was proposed by integrating state-of-the-art algorithms. A model named BioSemAF-BiLSTM (Biological Semantic Adaptive Fusion Bidirectional Long Short-Term Memory) was designed to more accurately capture essential features within protein sequences. It combined bioinformatics-derived sequence features and semantic representations of sequence subwords, employing a BiLSTM to extract global contextual information. Position-Specific Scoring Matrices (PSSMs) encode evolutionary information, while fastText-generated embeddings capture local semantics and biologically relevant residue patterns. To enhance feature representation, an attention-based Adaptive Feature Fusion (AF) module was used to integrate fastText embeddings with PSSM-derived numerical features, assigning dynamic weights to emphasize the most informative components. The fused features were then passed through fully connected layers for classification. Computational evaluation demonstrated that the proposed method significantly outperformed existing approaches in terms of sensitivity, specificity, and overall accuracy, highlighting the effectiveness of integrating biological embeddings, deep neural networks, and attention mechanisms for S-sulfenylation site prediction.

## 2 Materials and methods

**FIGURE 1:**
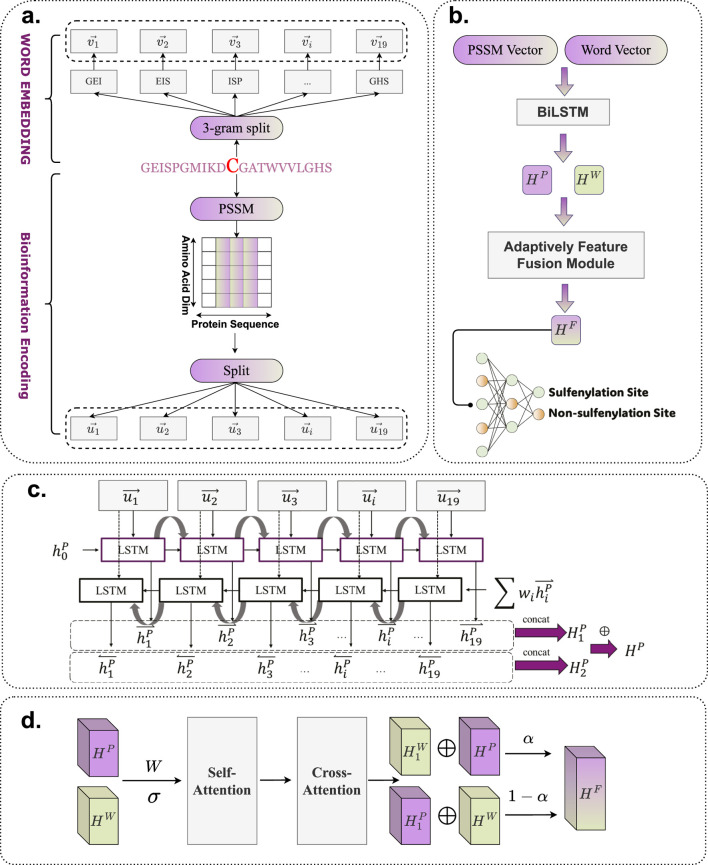
Flowchart of BioSemAF-BiLSTM. **(a)** Illustration of two types of sequence-based feature representations: semantic embeddings and bioinformational features. **(b)** Overview of the complete prediction pipeline: vectors from PSSM and fastText embeddings are processed by Bi-LSTMs, fused via the Adaptive Feature Fusion Module, and classified by a dense layer to predict S-sulfenylation sites. The illustration in this panel uses PSSM features as an example input. **(c)** Internal architecture of the Bi-LSTM module, where sequence representations are transformed into contextualized hidden features for downstream classification. **(d)** Workflow of the Adaptively Feature Fusion Module, which integrates semantic and evolutionary features into a unified representation; final site prediction is performed after this module as shown in panel **(b)**. Abbreviations: PSSM, Position-Specific Scoring Matrix; Bi-LSTM, Bidirectional Long Short-Term Memory.

The detailed architecture of BioSemAF-BiLSTM is illustrated in Figure 1, comprising four main components: Word Embedding, Bio-information Encoding, Bi-LSTM, and the Adaptive Feature Fusion Module. A fully connected dense layer is appended at the end to perform binary classification. This architecture is designed to leverage the complementary strengths of all modules, thereby enhancing prediction accuracy and model robustness.

### 2.1 Benchmark dataset

We utilized the dataset developed in the iSulf-Cys framework (Xu et al., 2016), which is based on the work of Yang et al. (2014). The dataset comprises 7,124 non-sulfenylation cysteine sites and 1,045 sulfenylation cysteine sites. In total, the data are derived from 778 protein sequences and include 1,105 sulfenylation cysteine sites. The corresponding protein sequences are available at https://ndownloader.figstatic.com/files/5004001.

For each cysteine site subjected to analysis, Xu et al. constructed peptide samples by extracting the 10 amino acids upstream and downstream, resulting in a sequence window of size 21 centered on the target cysteine. If the surrounding sequence contained fewer than 10 residues, the placeholder residue X was used to pad the sequence. The representation of a sample is as Equation 1:

$$\text{Peptide}=A_{-10}A_{-9}\ldots CA_{1}A_{2}A_{3}\ldots A_{10}$$ (1)

where $A_{i}\in \left\{20\text{ amino acids}\right\}\cup \left\{X\right\}$ , and i range from −10 to 10, representing the one-dimensional coordinate relative to the central cysteine. A peptide segment with a central cysteine that is a sulfenylation site is defined as a positive sample, while all others are classified as negative samples. To minimize redundancy and reduce homology bias, peptide segments sharing more than 40% sequence similarity were removed from the dataset. Ultimately, 145 positive and 268 negative samples were randomly selected as the test set, while the remaining 900 positive and 6,858 negative samples were allocated to the training set. A summary of the dataset is presented in Table 1. This dataset has been widely adopted in sulfenylation site prediction studies (Ju and Wang, 2018; Khan and Pi, 2020; Do et al., 2020), and it exhibits a pronounced imbalance between positive and negative classes.

**TABLE 1:** The number of positive and negative samples in training and independent test dataset.

Data	Positive	Negative
Training Set	900	6,856
Test Set	145	268

### 2.2 Feature extraction

In this study, two strategies were considered for processing peptide segment features. First, bioinformatics-based features were introduced by encoding sequences using the Position-Specific Scoring Matrix (PSSM). Second, natural language processing techniques were applied to generate word embeddings from protein sequences, thereby enabling the model to fully capture the latent semantic information embedded within the sequences. This approach has been validated as effective in previous study (Do et al., 2020).

#### 2.2.1 PSSM

PSSM, or Position-Specific Scoring Matrix, is a bioinformatic representation used to characterize evolutionary conservation in biological sequences. Evolutionarily conserved amino acid residues are often associated with functional motifs that play critical roles in protein functions, including post-translational modifications (PTMs) (Li and Dohlman, 2022). Therefore, PSSM serves as a valuable bioinformatic feature for representing peptide segments in prediction models.

PSSM describes and quantifies the distribution of amino acids (or nucleotides) at each position within a sequence, reflecting both evolutionary conservation and mutational tendencies. It has been widely applied across a range of site prediction tasks. For instance, Yuan et al. (2021) introduced PSSM as a key feature in characterizing protein–protein interaction (PPI) nodes. In PTM site prediction, PSSM has also shown substantial utility. PSSM-Suc employs PSSM to encode succinylation sites for predictive modeling (Dehzangi et al., 2017), whereas PSSM-Sumo utilizes a variant called PsePSSM to encode SUMOylation sites (Khan et al., 2024). Both approaches demonstrated strong predictive performance, further validating the effectiveness of PSSM-based representations.

A standard PSSM has a matrix dimension of 20 rows—each corresponding to one of the 20 standard amino acids—and a number of columns equal to the length of the peptide or sequence. The canonical format of the PSSM is defined as Equation 2:

$$\text{PSSM}=\left(\begin{array}{cccc} a_{11} & a_{12} & \ldots & a_{1L}\\ a_{21} & a_{22} & \ldots & a_{2L}\\ \vdots & \vdots & \ddots & \vdots \\ a_{20,1} & a_{20,2} & \ldots & a_{20,L} \end{array}\right)$$ (2)

Where $a_{ij}$ represents the probability of the *i*th amino acid mutating to another amino acid at the *j*th position, with i ranging from 1 to 20 (representing the amino acid types) and j ranging from 1 to L (representing the sequence length, with L being the total length of the sequence).

In this study, PSSMs were generated using Position-Specific Iterated BLAST (PSI-BLAST; https://blast.ncbi.nlm.nih.gov/Blast.cgi) against the NCBI non-redundant (NR) protein database (Altschul et al., 1997). Following common practice (Nie et al., 2017), we used three iterations with an expectation value (E-value) of 0.001. It should be noted that PSI-BLAST was used solely to perform iterative alignment for PSSM generation, not for *de novo* homologous sequence search. Other multiple-sequence-alignment tools such as HHblits (Remmert et al., 2011) or Clustal Omega (Sievers and Higgins, 2014) could also be applied for this purpose, but PSI-BLAST remains widely used in related work.

### 2.2.2 Word embedding

Language is one of the most intuitive forms of representation, and humans have long sought to describe the natural world through linguistic expressions. By assigning specific letters to amino acids, protein sequences can be directly encoded as character strings. These sequences contain rich semantic content that can be mined using appropriate natural language processing (NLP) techniques. This section focuses on local protein-related tasks, particularly on predicting residue-level features from sequence data. Window-based models have proven effective in various PTM site prediction tasks. For instance, Brandes et al. (2015) introduced a framework that predicts PTM sites by segmenting protein sequences into fixed-size windows, highlighting the potential of semantic features in protein feature engineering.

With the continuous advancement of NLP technologies (Vaswani et al., 2017; Devlin et al., 2018), Word2Vec has emerged as a major breakthrough in the field (Mikolov et al., 2013a). By leveraging contextual information, it generates continuous low-dimensional vector representations for words (or characters), capturing latent semantic relationships embedded in language. This advancement has inspired novel approaches to protein sequence analysis. FastText, an enhanced version of Word2Vec, further expands upon this idea (Joulin et al., 2016). Compared to Word2Vec, fastText introduces subword modeling, which effectively captures internal morphological and structural features of words. This is particularly suitable for protein sequence modeling, as such sequences frequently contain variable and localized motifs. FastText not only captures local *n*-gram-like information during training but also addresses the Out-of-Vocabulary (OOV) problem—a critical advantage when analyzing complex biological sequences.

The core idea of fastText is to enhance traditional word embeddings by incorporating subword modeling. In contrast to the Word2Vec model, which assigns a distinct vector to each word, fastText represents a word as a combination of its constituent subwords, thereby capturing subtle internal variations within words. For example, the word *protein* can be split into subwords such as *pro*, *ote*, and *ein*, all of which participate in the model’s training. By encoding and embedding these subwords, fastText learns more nuanced semantic representations, making it especially effective in handling diverse and irregular protein sequences.

From a mathematical standpoint, fastText is built upon the Skip-gram architecture, where the objective is to maximize the conditional probability of predicting a target word given its surrounding context (Mikolov et al., 2013b). Given a training corpus composed of word sequences $w_{1},w_{2},\ldots ,w_{T}$ , the objective function of fastText is to learn embeddings for both words and their subwords by minimizing the following loss function (Equation 3):

$$L=-\overset{T}{\underset{t=1}{\sum }}\underset{-c\leq j\leq c,j\neq 0}{\sum }\log p\left(w_{t+j}|w_{t}\right)$$ (3)

where $w_{t}$ is the current word, $w_{t+j}$ is the context word, c is the size of the context window, and $p\left(w_{t+j}|w_{t}\right)$ is the conditional probability of predicting the target word $w_{t+j}$ . In fastText, $p\left(w_{t+j}|w_{t}\right)$ is no longer a simple word’s conditional probability but a conditional probability of the subwords. Specifically, for each word w , it is split into several subwords $s_{1},s_{2},\ldots ,s_{k}$ , and the probability of the target word is calculated as Equation 4:

$$p\left(w_{t+j}|w_{t}\right)=\underset{s\in S\left(w_{t+j}\right)}{\prod }p\left(s|w_{t}\right)$$ (4)

where S(w) represents the set of subwords for the word w , and $p\left(s|w_{t}\right)$ denotes the conditional probability of subword s given the context word $w_{t}$ .

In practical applications, fastText represents the semantic content of a word through both its own embedding and the embeddings of its constituent subwords. For protein sequences, dipeptides or tripeptides derived from amino acid residues can be regarded as subwords. Using this representation, fastText can convert short fragments of protein sequences—such as amino acid pairs or triplets—into vector embeddings, capturing the underlying contextual relationships between substructures. In this study, each peptide sequence is decomposed into 3-g as subwords. For a sequence consisting of n amino acids, this yields n−2 trigrams, and hence, generates n−2 corresponding word vectors that embed the sequence’s implicit order and semantic content. This process constitutes the fastText-based protein sequence embedding used in this study.

### 2.3 Resampling

As described in Section 2.1, the dataset used in our study exhibits a pronounced imbalance between positive and negative samples, necessitating the application of resampling techniques. Common resampling strategies include down-sampling and up-sampling. Down-sampling involves reducing the number of majority class instances to achieve a balanced class distribution, whereas up-sampling increases the number of minority class samples to the same effect. To minimize information loss from the sample space and retain protein residue-level features, up-sampling is prioritized. Synthetic Minority Over-sampling Technique (SMOTE) is one of the most widely used up-sampling methods (Chawla et al., 2002). Numerous variants of SMOTE have been proposed in subsequent studies, and this study adopts Support Vector Machine Synthetic Minority Over-sampling Technique (SVMSMOTE) for up-sampling (Demidova and Klyueva, 2017). The resampling procedure is outlined as follows.

1. Train the SVM model: First, a support vector machine (SVM) classifier is trained using minority class samples to determine the decision boundary. By maximizing the margin between classes, SVM effectively learns to delineate the optimal separation boundary for the training data.
2. Identify support vectors: Support vectors are those training samples situated closest to the decision boundary. These points are critical in defining the classification margin. SVMSMOTE leverages these support vectors to guide the generation of new synthetic instances.
3. Generate synthetic samples: For each minority class support vector, SVMSMOTE generates synthetic samples by interpolating between the support vector and its nearest neighbors. This process is similar to SMOTE, and the interpolation formula is given as Equation 5:

$$x_{\text{new}}=x_{\text{sv}}+\lambda \cdot \left(x_{\text{neigh}}-x_{\text{sv}}\right)$$ (5)

where $x_{\text{new}}$ is the generated synthetic sample, $x_{\text{sv}}$ is the current support vector, $x_{\text{neigh}}$ is its nearest minority class neighbor, and λ is a random number in the range [0, 1] that determines the interpolation proportion.

4. Generate the required number of synthetic samples: Based on the method above, SVMSMOTE generates enough synthetic samples to achieve a balanced class distribution in the training dataset.

For a schematic illustration of the SMOTE and SVMSMOTE procedures, please refer to Supplementary Figure S1.

### 2.4 Bi-LSTM

Considering the sequential nature of the two feature spaces used for protein representation in this study, a Bi-LSTM-based approach was adopted. The strength of LSTM in handling protein sequences lies in its capability to effectively capture long-range dependencies. In protein prediction tasks, especially in the identification of post-translational modification (PTM) sites, long-range contextual dependencies are critical (Shi et al., 2025; Vaswani et al., 2017) as relevant information in protein sequences often spans beyond local neighborhoods. Accurate functional prediction therefore, relies on a model’s ability to extract such extended context. Traditional recurrent neural networks (RNNs) struggle in these scenarios due to challenges such as vanishing and exploding gradients, which hinder learning across long temporal sequences. In contrast, Bi-LSTM incorporates both forward and backward contextual information along the sequence, allowing it to capture bidirectional dependencies that are essential for accurate PTM site prediction.

A standard Bi-LSTM consists of a forward LSTM and a backward LSTM. Specifically, the LSTM architecture introduces memory cells that determine whether to retain or discard information from past inputs. These memory cells are regulated by three gates: the input gate, forget gate, and output gate. The input gate controls how much of the current input is stored in the memory cell, the forget gate decides which parts of the previous memory to discard, and the output gate determines the information to be passed to the next layer in the network.

In this study, Bi-LSTM is employed as the core model to process protein sequence data. By combining the forward and backward passes, Bi-LSTM effectively captures bidirectional contextual information within the protein sequence, thereby enhancing the accuracy of sequence-based predictions.

Let f denote the forward LSTM process and b denote the backward LSTM process. For each time step t , the forward LSTM computes the following Equations 6‐10:

$$i_{t}=\sigma \left(W_{i}\cdot \left[h_{t-1}^{\left(f\right)},x_{t}\right]+b_{i}\right)$$ (6)

$$f_{t}=\sigma \left(W_{f}\cdot \left[h_{t-1}^{\left(f\right)},x_{t}\right]+b_{f}\right)$$ (7)

$$o_{t}=\sigma \left(W_{o}\cdot \left[h_{t-1}^{\left(f\right)},x_{t}\right]+b_{o}\right)$$ (8)

$$c_{t}^{\left(f\right)}=f_{t}\cdot c_{t-1}^{\left(f\right)}+i_{t}\cdot \tanh\left(W_{c}\cdot \left[h_{t-1}^{\left(f\right)},x_{t}\right]+b_{c}\right)$$ (9)

$$h_{t}^{\left(f\right)}=o_{t}\cdot \tanh\left(c_{t}^{\left(f\right)}\right)$$ (10)

Where σ is the sigmoid activation function, tanh is the hyperbolic tangent function, $x_{t}$ is the current input, $h_{t-1}^{\left(f\right)}$ is the previous time step’s hidden state, and $c_{t-1}^{\left(f\right)}$ is the previous time step’s memory state.

The backward LSTM operates similarly to the forward LSTM but processes the sequence in reverse order. In the standard Bi-LSTM model, the outputs of both the forward and backward LSTMs are combined to calculate the final output for each time step. However, in this study, the initial hidden state of the backward LSTM is modified. Specifically, the initial hidden state of the backward LSTM is set as a weighted sum of the forward LSTM’s initial hidden state, with the weights being trainable. This modification is expressed as Equation 11:

$$h_{0}^{\left(b\right)}=\sigma \left(W_{i}h_{i}^{\left(f\right)}\right)$$ (11)

Where $w_{i}$ is the weight for the i -th step of the forward LSTM, and $h_{i}$ is the hidden state at the i -th step. This improvement enables the backward pass to integrate information learned from the forward pass, enhancing backward propagation.

Additionally, the model incorporates residual connections, which can be expressed as Equation 12:

$$h_{t+1}^{\left(f\right)}=\alpha \cdot h_{t+1}^{\left(f\right)}+\left(1-\alpha \right)\cdot h_{t}^{\left(f\right)}$$ (12)

Where α is a hyperparameter that adjusts the weighting between the current hidden state and the previous hidden state. The residual connection retains information from the previous time step’s hidden state, enhancing the model’s ability to capture long-range dependencies.

### 2.5 Adaptively feature fusion module

Feature extraction in this study is based on both bioinformatics features and semantic features derived from protein sequence fragments. These two types of features emphasize different aspects of information representation: bioinformatics features highlight the structural and functional features of the sequence, whereas semantic features capture contextual relationships at a natural language level. However, due to potential redundancy or inconsistency between these two modalities, an Adaptive Feature Fusion Module is proposed to integrate and utilize them more effectively. This module is designed with two primary objectives: first, to accurately identify and eliminate redundant information to prevent irrelevant or noisy features from impairing model performance; and second, to enhance discriminative features that are crucial to the target task, thereby improving both the learning efficiency and predictive accuracy of the model. The Adaptive Feature Fusion Module not only manages the allocation of importance across different feature types, but also dynamically adjusts the fusion strategy to accommodate variations across different tasks or data distributions.

This study proposed a fusion module leveraging both self-attention and cross-attention mechanisms. The goal of this module is to dynamically assign feature weights using these attention mechanisms, thereby extracting salient information and achieving efficient fusion. A detailed introduction to this module is provided below.

Let the features to be fused be $H^{P}$ (size $N\times d_{1}$ ) and $H^{W}$ (size $N\times d_{2}$ ). Since the dimensions of $H^{P}$ and $H^{W}$ may differ, projection layers were initially established to map both features into a common dimensionality d . This is done via linear projection followed by a non-linear activation function σ (Equations 13, 14):

$$H_{1}^{P}=\sigma \left(W_{1}H^{P}\right)$$ (13)

$$H_{1}^{W}=\sigma \left(W_{2}H^{W}\right)$$ (14)

where $W_{1}\in R^{d\times d_{1}}$ and $W_{2}\in R^{d\times d_{2}}$ .

Next, self-attention is applied to both $H_{1}^{P}$ and $H_{1}^{W}$ . Self-attention is a mechanism used to compute the relevance of each element in the sequence to every other element, allowing it to capture global context. It dynamically assigns higher weights to important elements in the sequence, thus improving the model’s ability to understand complex dependencies. The calculation of Self-attention is as Equation 15:

$$\text{Self}-\text{Attention}\left(Q,K,V\right)=\text{softmax}\left(\frac{QK^{T}}{\sqrt{d}}\right)V$$ (15)

where Q , K , and V are linear transformations of $H_{1}^{P}$ and $H_{1}^{W}$ . Specifically, self-attention is applied to extract their respective key feature patterns (Equations 16, 17):

$$H_{2}^{P}=\text{Self}-\text{Attention}\left(H_{1}^{P}\right),$$ (16)

$$H_{2}^{W}=\text{Self}-\text{Attention}\left(H_{1}^{W}\right),$$ (17)

Next, $H_{2}^{P}$ and $H_{2}^{W}$ are combined using the cross-attention mechanism to model the interaction between the two types of features. Cross-attention extracts important information by focusing on the most relevant parts of one feature set relative to another. By doing so, it effectively fuses features from different sources and highlights key associations between them. The computation of Cross-attention is similar to Self-attention, but the design of Q , K , and V differs (Equation 18).

$$\text{Cross}-\text{Attention}\left(Q,K,V\right)=\text{softmax}\left(\frac{QK^{T}}{\sqrt{d}}\right)V$$ (18)

Here, Q comes from the linear transformation of one feature set, while K and V come from the linear transformations of the other feature set. Therefore, relevant semantic feature information can be extracted from the bioinformatics features, as expressed by Equations 19, 20:

$$H_{PW}=\text{Cross}-\text{Attention}\left(H_{2}^{P},H_{2}^{W}\right),$$ (19)

$$H_{WP}=\text{Cross}-\text{Attention}\left(H_{2}^{W},H_{2}^{P}\right),$$ (20)

Finally, we perform a weighted fusion of the outputs from both Self-attention and Cross-attention (Equation 21):

$$H^{F}=\alpha \left(H_{PW}+H_{2}^{W}\right)+\left(1-\alpha \right)\left(H_{WP}+H_{2}^{P}\right)$$ (21)

where α is a weight parameter. The fused feature representation, $H^{F}$ , is then passed to the subsequent dense layers.

Because positional biases around modified cysteines are supported by redox chemistry and chemoproteomics (Gupta and Carroll, 2014; Huang et al., 2019), the attention weights in the Adaptive Feature Fusion module serve as a proxy for residue-level importance within the ±10 window, encouraging the model to up-weight positions consistent with these biologically plausible contexts.

### 2.6 Cross-validation and performance evaluation

To evaluate the robustness and generalization capability of our proposed method, we employed a repeated 10-fold cross-validation strategy. Specifically, the entire dataset was randomly partitioned into 10 folds, of which 9 folds were used for training and one fold for testing. Similar to other studies, we adopted commonly used evaluation metrics, including the Area Under the Receiver Operating Characteristic Curve (auROC), Area Under the Precision-Recall Curve (auPRC), Sensitivity (Sn), Specificity (Sp), Accuracy (ACC), and Matthews Correlation Coefficient (MCC), to comprehensively assess the model’s predictive performance. The formulas for the latter four metrics are as Equations 22–25:

$$\text{Sn}=\frac{TP}{TP+FN}$$ (22)

$$\text{Sp}=\frac{TN}{TN+FP}$$ (23)

$$\text{ACC}=\frac{TP+TN}{TP+TN+FP+FN}$$ (24)

$$\text{MCC}=\frac{TP\cdot TN-FP\cdot FN}{\sqrt{\left(TP+FP\right)\left(TP+FN\right)\left(TN+FP\right)\left(TN+FN\right)}}$$ (25)

Where TP , TN , FP , and FN represent the true positives, true negatives, false positives, and false negatives, respectively. Since we use an imbalanced dataset, it is highly appropriate to include auROC and auPRC as evaluation metrics. auPRC better reflects the model’s performance on the positive class, especially in situations of class imbalance (Hancock et al., 2023).

Beyond model performance assessment, we quantitatively evaluate feature adequacy using a sequence compression–based approximation of Kolmogorov complexity (KC) (Li and Vitányi, 1997). In plain terms, Kolmogorov complexity asks “what is the shortest description that can reproduce a given sequence?“. If the feature representation preserves most of the regularities and patterns in the raw sequence, then compressing the features (or the features concatenated with the raw sequence) will be nearly as effective as compressing the raw sequence itself. Therefore, a small estimated information-loss rate indicates that the features retain most sequence-level biochemical signals relevant to prediction, whereas a large loss suggests that important patterns may have been discarded. The information-loss rate $\eta_{\text{loss}}$ is defined as Equation 26:

$$\eta_{\text{loss}}=\frac{K\left(X|f\left(X\right)\right)}{K\left(X\right)}=\frac{K\left(X,f\left(X\right)\right)-K\left(f\left(X\right)\right)}{K\left(X\right)}.$$ (26)

Here K(X) denotes the (prefix) Kolmogorov complexity of a string X , formally (Equation 27)

$$\begin{array}{c} K\left(X\right)=\min\{\;|p|:\text{ a program }p\text{ outputs }X\\ \text{on a fixed universal }\text{Turing machine }U\;\}. \end{array}$$ (27)

In words, K(X) is the length (in bits) of the shortest program that generates X on U . This definition is invariant up to an additive constant (independent of X ) due to the invariance theorem (Li and Vitányi, 1997). Likewise, K(X,f(X)) is the joint complexity of the concatenated description of X and f(X) , and K(X∣f(X)) is the conditional complexity (the additional information needed to reconstruct X given f(X) ).

In this study, X denotes the raw amino-acid sequence fragment and f(X) the feature-extraction mapping (fastText subword embeddings and the PSSM). Since exact Kolmogorov complexity is non-computable (due to the undecidability of the halting problem) (Li and Vitányi, 1997), we approximate it using off-the-shelf lossless compressors, which provide practical upper bounds to K . Concretely, let C(⋅) be the compressed length (bytes) under a fixed ZIP/DEFLATE compressor; we estimate (Equation 28)

$$\hat{K}\left(X\right)=C\left(X\right),\hat{K}\left(f\left(X\right)\right)=C\left(f\left(X\right)\right),\;\hat{K}\left(X|f\left(X\right)\right)\approx C\left(f\left(X\right)\|X\right)-C\left(f\left(X\right)\right),$$ (28)

where ‖ denotes concatenation with a delimiter to avoid boundary artifacts. This estimator is closely related to the normalized compression distance framework (Cilibrasi and Vitányi, 2005). For biological symbol sequences, compression-based complexity estimates—often based on Lempel-Ziv variants—have a long history and have been successfully applied to genetic texts (Gusev et al., 2000).

This metric is particularly crucial for our sulfenylation site prediction task where we process 21-residue sequence fragments without structural information. Given that our features convert symbolic sequences to numerical representations for BiLSTM classification, quantifying information preservation ensures the extracted features maintain biochemically relevant patterns while reducing noise (Zvonkin and Levin, 2007; Kolmogorov, 1983).

## 3 Results and discussions

### 3.1 Sample sequence content analysis

To better illustrate the differences between sulfenylation site samples and non-sulfenylation site samples at the residue level, this study employed the Two-Sample Logo technique. This method utilizes statistical analysis to identify significant distinctions between central cysteine residues and their surrounding amino acids in positive and negative samples (Schneider and Stephens, 1990; Crooks et al., 2004). The Two-Sample Logo is a commonly used visualization tool in sequence content analysis for various PTMs, offering clear insights into amino acid distribution patterns around the modification site and serving as explanatory support for site prediction.

**FIGURE 2:**
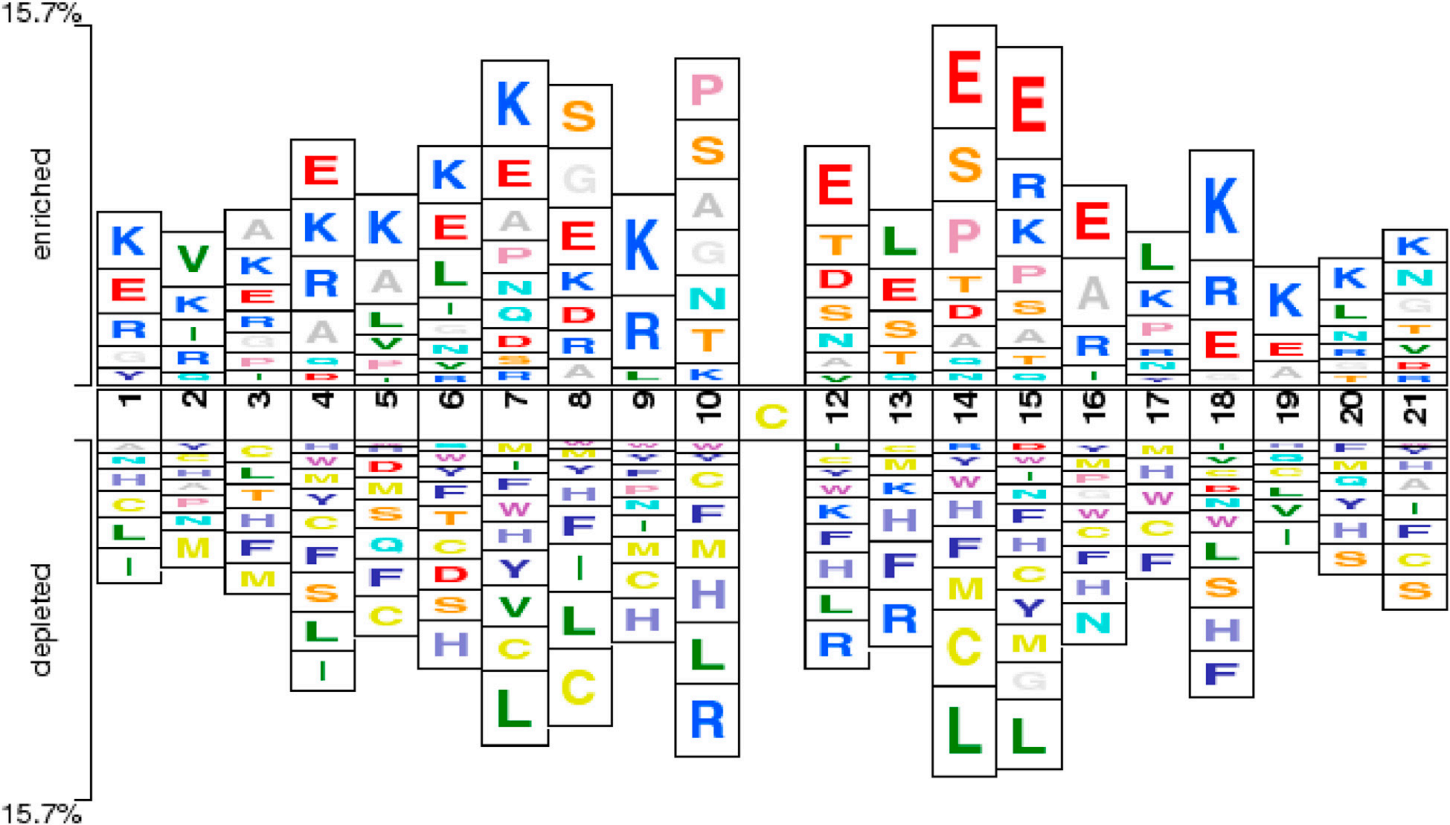
Two-Sample Logoshowing position-specific enrichment and depletion of amino acids around the central cysteine (Cys) residue. The horizontal axis indicates sequence positions within a 21-residue window, centered on the target Cys at position 11. The vertical axis represents enrichment or depletion levels of amino acids at each position. Enrichment of charged residues proximal to the central Cys aligns with prior mechanistic and chemoproteomic evidence showing that local electrostatics modulate thiol pKa and sulfenic-acid reactivity (Gupta and Carroll, 2014).

Figure 2 depicts the Two-Sample Logo comparison between the positive and negative sample sets used in this study, with a significance threshold (P-value) set to 0.5. The figure reveals notable differences between multiple positive and negative samples. For instance, non-central cysteine residues are more prevalent in negative samples, particularly at positions +1, +4, +5, +6, +7, +8, +9, +10, +14, +15, +17, and +21. This observation is consistent with the findings of the Bi-directional Gated Recurrent Unit network with Self-Attention (BiGRUD-SA) model, despite differences in datasets, highlighting the strong generalization capability of the BioSemAF-BiLSTM model proposed in this study (Zhang et al., 2023). Moreover, other amino acids also exhibit statistically significant differences between the two sample groups, such as.

• Lysine (K): Frequently appears in positive samples at positions +1, +3, +4, +5, +6, +7, +9, +15, +18, +19, +20;

• Glutamic acid (E): Frequently appears in positive samples at positions +1, +4, +6, +7, +8, +12, +13, +14, +15, +16, +18;

• Histidine (H): Frequently appears in negative samples at positions +3, +6, +7, +8, +9, +10, +12, +13, +14, +15, +16, +17, +18, +20.

Importantly, the enrichment of charged residues such as Lys (K) and Glu (E) flanking the central cysteine is consistent with established redox chemistry and chemoproteomic observations: local electrostatics and hydrogen-bonding networks modulate cysteine thiol pKa and the lifetime of sulfenic acid, biasing oxidation toward specific sequence microenvironments (Gupta and Carroll, 2014). Large-scale surveys likewise report redox-sensitive cysteines occurring in characteristic local contexts across proteomes (Huang et al., 2019; Fu et al., 2023; Akter et al., 2018). This concordance provides biological support for the sequence patterns highlighted by our Two-Sample Logo and for subsequent sequence-based prediction.

These features capture significant distinctions between positive and negative samples at the corresponding P-values, thereby confirming the reliability and effectiveness of the sample data and laying a robust foundation for further research.

Additionally, according to Do et al. (2020), no definitive correlation has been established between sulfenylation sites and their surrounding amino acid compositions. However, investigating its biological significance remains crucial. Mu et al. (Mu et al., 2024) further elucidated the mechanisms underlying protein sulfenylation. They highlighted that variations in the generation and distribution of intracellular oxidants (e.g., hydrogen peroxide) lead to differential oxidant levels across organelles and subcellular compartments, thereby influencing sulfenylation occurrence. The pKa of the cysteine thiol group is significantly modulated by protein structure, including its secondary and tertiary structures, hydrogen bonding, macrodipoles, and electrostatic interactions. Lower pKa values enhance the nucleophilicity of the thiol group, rendering it more susceptible to sulfenylation (Kortemme and Creighton, 1995; Ferrer-Sueta et al., 2011). Additionally, the polarization of the thiol group and the non-bonding electrons of adjacent atoms directly modulate its nucleophilicity, thereby affecting sulfenic acid formation.

These studies offer critical biological insights into the sequence-based relationship between sulfenylation sites and their surrounding amino acids. They also provide theoretical support for model development and optimization, further advancing our understanding of sulfenylation mechanisms.

### 3.2 Parameter tuning experiment

To ensure optimal model performance, we performed an extensive tuning process for the key hyperparameters of the BioSemAF-BiLSTM model. These hyperparameters include *n*-gram subword length (Length), fastText word vector dimensionality (Dim), and the number of attention heads in the adaptive feature fusion module (Head). For each type of parameter, values were progressively adjusted within reasonable ranges, with appropriate step sizes set to precisely identify local optima. Subsequently, by integrating the optimal settings across multiple parameters, the global best configuration for overall model performance was determined.

Table 2 illustrates the effects of varying parameter configurations on model performance, including *n*-gram subword lengths (2, 3, 4), fastText embedding dimensions (30, 60, 90), and the number of attention heads in the adaptive feature fusion module (3, 6, 9). The evaluation metrics used in the analysis include ACC, MCC, Sn, and Sp. After individually tuning each hyperparameter and evaluating the overall performance, the optimal settings for these three key parameters were determined as follows.

• *n*-grams Subword Length: Selected as 3, dividing the protein sequence into subwords with three amino acids as one unit;

• fastText Word Vector Dimensionality: Determined to be 60, while keeping the other two hyperparameters at their optimal values;

• Number of Attention Heads in the Adaptive Feature Fusion Module: Determined to be 3.

**TABLE 2:** Performance of the BioSemAF-BiLSTM model under different hyperparameter settings.

Hyperparameters	Value	Sn	Sp	ACC	MCC
Length	2	91.44	85.14	88.24	0.7100
	**3**	**93.52**	**87.18**	**89.26**	**0.7000**
	4	92.61	84.13	86.69	0.6800
Dim	30	89.29	82.58	85.33	0.6500
	**60**	**94.34**	**84.95**	**89.14**	**0.7100**
	90	89.98	82.76	86.67	0.7000
Head	**3**	**90.47**	**83.28**	**88.95**	**0.7000**
	6	87.89	82.61	86.35	0.6800
	9	87.52	81.45	85.82	0.6800

Through analyzing the impact of different parameters on model performance during the debugging process, the following conclusions were drawn.

1. The length of *n*-grams subwords significantly affects the model’s feature representation ability. Too short may lead to insufficient feature information, while too long may introduce irrelevant contextual information, thus affecting model performance.
2. The change in fastText word vector dimensions mainly affects the semantic expression accuracy of input features. A 60-dimensional vector performs best in balancing computational cost and performance improvement.
3. Increasing the number of attention heads can enhance the feature fusion module’s ability to capture multimodal features. However, too many heads can increase model complexity and potentially lead to overfitting.

The final experimental results validated the effectiveness of these hyperparameter configurations, laying the foundation for future model applications.

### 3.3 Comparison of prediction performance of different models

To comprehensively assess the predictive performance of BioSemAF-BiLSTM, we conducted experiments from two perspectives: data resampling strategies and classifier performance evaluation. To mitigate the issue of data imbalance, multiple up-sampling and down-sampling techniques were explored to enhance the model’s classification performance. Results indicate that SVMSMOTE significantly improves the model’s generalization ability while preserving the original feature distribution, making it the preferred resampling method for this study. Table 3 summarizes the experimental outcomes of various sampling techniques.

**TABLE 3:** Experimental results of different data resampling methods.

Method	Algorithm	Sn	Sp	ACC	MCC
Up-sampling	SMOTE	80.96	69.98	77.43	0.5900
	RandomOverSampler	78.06	67.26	77.64	0.6400
	KMeansSMOTE	88.28	82.49	86.38	0.7000
	SVMSMOTE	89.66	84.22	89.02	0.6800
Down-sampling	TomekLinks	68.27	61.42	62.88	0.3100
	NearMiss	60.92	63.81	63.2	0.3200

**FIGURE 3:**
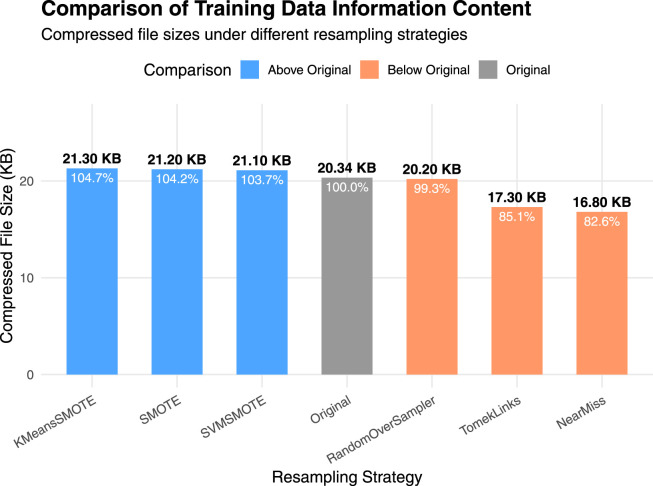
Comparison of training data information content under different sampling strategies. Bars represent the compressed file sizes (in KB) of the original, upsampled, and downsampled training datasets after applying gzip compression. A larger compressed size indicates higher algorithmic information content.

It can be seen that there exists a strong gap between the results of the up-sampling and down-sampling methods. To further interpret the significant performance differences observed between them, we conducted a supplementary experiment using a compression-based approximation of Kolmogorov complexity to quantify the information content of the training data under different sampling strategies. Specifically, the original, upsampled, and downsampled training sets were each saved in CSV format and compressed using the gzip algorithm. For practical estimation, we used gzip as a standard lossless compressor to approximate Kolmogorov complexity. Alternative strategies, such as reduced-alphabet encodings or specialized compressors designed for biological sequences (Gusev et al., 2000), could yield slightly different absolute compression sizes, but the relative differences between sampling strategies remain robust. Since Kolmogorov complexity is incomputable in general, the size of the compressed file provides a practical estimate of the dataset’s algorithmic information content. Figure 3 shows the results.

It can be observed that the up-sampling methods produce gzip-compressed files with sizes similar to that of the original dataset. This is because the new samples generated during up-sampling are typically derived from the existing data distribution through interpolation or duplication. Although the number of samples increases, the redundancy in the data also rises, which limits any substantial increase in information complexity. In contrast, down-sampling directly reduces the number of samples, potentially discarding minority class or boundary instances, which results in a significant loss of information. Consequently, the compressed file sizes for down-sampling methods are noticeably smaller than those of the up-sampling methods, indicating a reduction in information complexity.

This finding provides an information-theoretic justification for the performance differences reported in the main experiments: down-sampling reduces the dataset’s descriptive richness and diversity, thereby impairing the model’s generalization capability, whereas up-sampling maintains the complexity while improving class balance (Gao et al., 2024; Thabtah et al., 2020).

**FIGURE 4:**
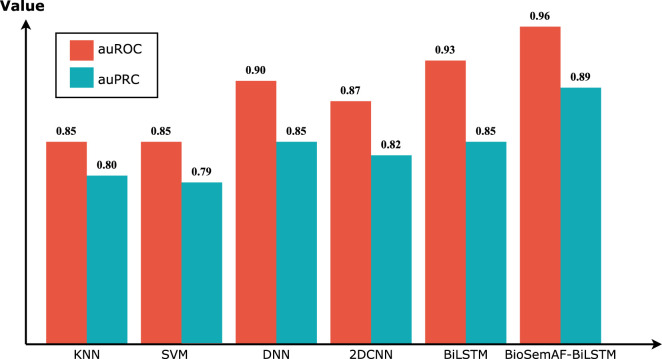
AuROC and auPRC of different classifiers.

In the classifier performance comparison experiment, we selected traditional machine learning algorithms (such as KNN, SVM) and other deep learning models (such as DNN, 2DCNN, BiLSTM) for comparative testing. To ensure fairness, the hyperparameters of each model were adjusted to optimal values. Specifically, the k value of KNN was set to 10, and the penalty coefficient C for SVM was set to 2, consistent with Do’s study (Do et al., 2020). DNN used a four-layer fully connected network, with dimensions of 256, 128, 64, and 32 for each layer. BiLSTM consisted of two layers of bidirectional long short-term memory units, followed by three fully connected layers. 2DCNN included two convolutional layers, two average pooling layers, and one fully connected output layer, with Sigmoid used as the activation function. In these settings, the models were compared according to the auROC and auPRC metrics, with the results shown in Figure 4. The experimental results demonstrate that BioSemAF-BiLSTM performs excellently on both of these key metrics, with an auROC of 0.96, higher than DNN (0.90), BiLSTM (0.93), and 2DCNN (0.87). Its auPRC also reached 0.89, further validating the model’s ability to accurately predict approximately 89% sulfenylation sites.

Comprehensive analysis indicates that the outstanding performance of BioSemAF-BiLSTM can be primarily ascribed to two key factors. Firstly, SVMSMOTE effectively balances the ratio of positive to negative samples, thereby ensuring more robust input data. Secondly, integrating bidirectional long short-term memory units with the adaptive feature fusion module enables the model to comprehensively capture contextual information within protein sequences, leading to enhanced feature learning and classification performance in comparison with conventional machine learning algorithms and other deep learning architectures. These findings underscore the high reliability and generalization capacity of BioSemAF-BiLSTM in sulfenylation site prediction, offering valuable insights for future research.

### 3.4 Comparison of prediction performance with different features

We employed a combination of word embedding techniques and sequence evolutionary conservation information to generate features, aiming to enhance the model’s predictive performance. Regarding word embedding techniques, fastText was compared with other feature representation methods, such as one-hot encoding and Global Vectors (GloVe) (Pennington et al., 2014). When all methods were evaluated under their respective optimal parameter settings, fastText demonstrated a clear advantage. This result suggests that fastText’s ability to utilize subwords plays a crucial role in improving the performance of neural networks, which is consistent with the findings reported by Do et al. (2020).

In the generation of features based on sequence evolutionary conservation information, this study compared PSSM with BLOSUM62 and amino acid composition (AAC). For BLOSUM62, the substitution scores corresponding to each residue were extracted from the matrix and arranged into per-residue 20-dimensional vectors, resulting in a feature representation of the same shape as the PSSM, which was then fed into the BiLSTM in the same way as the PSSM features. Similarly, under the condition that all parameters were set to optimal values, the PSSM features exhibited superior prediction performance.This finding further suggests a significant correlation between sequence evolutionary conservation information and sulfenylation sites.

The results are illustrated in Table 4. In conclusion, fastText’s subword technique and PSSM features have demonstrated their superiority in semantic feature extraction and biological information representation, respectively, providing a solid foundation for the model’s efficiency and accuracy. This not only validates the importance of multimodal feature generation but also offers strong reference for future research.

**TABLE 4:** Experimental results of different features and feature combinations.

Method	Sn	Sp	ACC	MCC
FastText	90.96	83.98	88.53	0.7100
GloVe	81.94	85.74	83.64	0.6300
One-hot	68.85	71.44	70.39	0.4200
PSSM	86.1	90.83	89.25	0.7200
BLOSUM62	77.81	83.87	82.38	0.5400
AAC	70.43	64.95	69.52	0.4300
FastText + PSSM	91.86	88.29	90.17	0.7500
GloVe + PSSM	87.52	87.40	87.46	0.7100
One-hot + PSSM	85.42	88.71	87.06	0.6300
FastText + BLOSUM62	89.48	86.32	88.00	0.7000
GloVe + BLOSUM62	85.36	86.91	86.18	0.6750
FastText + PSSM + GloVe	92.10	88.11	90.12	0.7400
FastText + PSSM + BLOSUM62	91.22	87.12	89.26	0.7400
All	92.34	87.91	90.65	0.7300

To further explore the potential complementarity between semantic and biological features, several fusion strategies were designed and evaluated. Among them, the combination of fastText and PSSM achieved promising performance, indicating that integrating diverse modalities enhances the model’s representation capability.

Notably, further improvements were observed when additional features such as GloVe and BLOSUM62 were included. For instance, the combination of fastText, PSSM, and GloVe achieved the Sn of 92.10, and integrating all six features yielded the best overall performance (ACC: 90.65, Sn: 92.34). These results demonstrate that while deeper feature fusion can be beneficial, the marginal gains may decrease due to redundancy. In addition, it is worth noting that feature fusion increases not only the input dimensionality but also the time cost of feature extraction and model training. In practice, the combination of fastText and PSSM offers a favorable trade-off between performance and computational efficiency.

### 3.5 Feature ablation experiment

In order to evaluate the impact of different feature information on model performance, we designed ablation experiments to observe the role of individual features in the prediction task. Table 5 presents the performance metrics when using only PSSM features and only word embedding features. The experimental results show that when using only PSSM features or only word vector features as inputs, the model’s prediction performance decreases. However, when both PSSM and word vector features are used together, the model’s metrics reach their optimal values.

**TABLE 5:** Performance comparison among single-feature inputs and fused-feature inputs. All values are reported as mean ± standard deviation and expressed in percentage.

Feature	Sn	Sp	ACC	MCC	$\eta_{\mathrm{loss}}$
Only word vector	76.52 ± 1.34	83.20 ± 2.49	78.39 ± 1.49	59.00 ± 2.41	21.00 ± 0.95
Only PSSM	80.16 ± 2.91	71.86 ± 1.37	77.28 ± 0.93	71.00 ± 1.69	28.00 ± 1.32
Both	90.74 ± 2.70	83.74 ± 2.74	89.32 ± 0.81	70.00 ± 2.49	11.00 ± 0.65

Bold values indicate the best performance in each column.

This conclusion indicates that information from a single aspect (such as sequence evolutionary conservation or semantic features) cannot fully represent the global statistical patterns of the data. Relying solely on one feature may overlook some key information. PSSM features capture the biological evolutionary conservation of protein sequences, revealing their potential functional importance, while word vector features, through subword modeling, effectively represent local semantic relationships in sequences. Therefore, combining bioinformatics features (PSSM) with semantic features (word vectors) allows for a more comprehensive integration of the biological significance and statistical properties of the sequence, significantly improving the model’s prediction ability. For the optimal combination of fastText and PSSM features, the estimated information loss during feature extraction is approximately 11%, which is considered acceptable for maintaining sequence-level biochemical relevance (Gusev et al., 2000). This supports the reliability of the extracted features for downstream sulfenylation site prediction. Through the ablation experiment, we further validated the necessity and effectiveness of multidimensional feature fusion, providing theoretical support for building more robust prediction models.

### 3.6 Comparison with other prediction tools

We compared the performance of the BioSemAF-BiLSTM model with existing prediction tools. To ensure the accuracy and fairness of the results, three state-of-the-art prediction tools were selected: iSulf-Cys, Sulf_FSVM, and fastSulf-DNN. These tools were trained and tested using the same dataset as in this study. Additionally, to maintain consistency with previous studies, we followed the standard evaluation protocol of the iSulf-Cys benchmark (Xu et al., 2016), reporting results from both repeated 10-fold cross-validation on the training set and evaluation on the independent gold-standard test set provided with iSulf-Cys. Within each training set, 10% of the data was further set aside as a validation subset for hyperparameter tuning and early stopping, ensuring that model optimization was not influenced by the final test fold. To reduce randomness due to single partitioning, the 10-fold cross-validation process was repeated 10 times with different random seeds.

These three tools use different feature generation methods and model architectures.

• fastSulf-DNN: Uses only biological subwords as features and adopts a relatively simple deep learning network architecture.

• iSulf-Cys: Uses physicochemical and distributional properties of amino acids as feature inputs.

• Sulf_FSVM: Combines mRMR feature selection techniques for feature optimization.

Table 6 reports the mean and standard deviation (±) of evaluation metrics in 10-fold CV and illustrates the results in independent test. The experimental results show that BioSemAF-BiLSTM outperforms all of these tools in all evaluation metrics. Specifically, on the MCC metric, it exceeds the next highest by about 17% on the independent test; on the ACC metric, it outperforms the next highest method by 12%; on the Sn metric, it surpasses the next highest method by 5%; and on the Sp metric, it exceeds the next highest method by 18%. Furthermore, despite integrating multidimensional features, the network architecture of BioSemAF-BiLSTM is not overly complex, indicating that the model design achieves improved performance while maintaining a certain level of simplicity.

**TABLE 6:** Comparison of BioSemAF-BiLSTM with three state-of-the-art prediction tools in repeated 10-fold cross-validation and on the independent gold-standard test set provided with iSulf-Cys.

Evaluation	Predictors	Sn	Sp	ACC	MCC
10-fold CV	iSulf-Cys	67.31 ± 1.85	63.89 ± 2.17	65.59 ± 1.63	0.3057 ± 0.028
	Sulf_FSVM	68.54 ± 2.03	68.03 ± 1.95	68.29 ± 1.77	0.3246 ± 0.030
	fastSulf-DNN	76.20 ± 1.65	83.20 ± 1.42	79.90 ± 1.33	0.6091 ± 0.022
	BioSemAF-BiLSTM (Proposed)	91.86 ± 1.07	88.29 ± 1.15	90.17 ± 0.98	0.7538 ± 0.014
Independent Test	iSulf-Cys	68.97	65.67	66.83	0.3300
	Sulf_FSVM	80.89	68.66	72.88	0.4700
	fastSulf-DNN	85.71	69.47	77.09	0.5600
	BioSemAF-BiLSTM (Proposed)	90.19	87.78	89.32	0.7362

## 4 Conclusion

We developed a prediction tool for identifying protein S-sulfenylation sites, aiming to fully extract informative features from protein sequences. Two main categories of features were constructed: one based on semantic biological subword embedding, utilizing fastText to capture local semantic relationships within protein sequences; the other based on sequence evolutionary conservation, using PSSM features to represent the functional conservation of proteins. After processing these two types of features with BiLSTM, they were integrated through an adaptive feature fusion module and subsequently embedded into a dense layer for classification. To validate the effectiveness of the model, various experiments were conducted, including parameter tuning, ablation studies, and comparative analysis with other prediction tools. Extensive experimental results demonstrated that the model exhibited significant performance advantages in ten-fold cross-validation and achieved a prediction accuracy of 89.32% on the independent test set, substantially outperforming existing state-of-the-art algorithms. This outcome strongly supports the robustness and generalization capability of the proposed model. Identifying sulfenylation sites is an essential step in deciphering protein functional regulation networks. This study not only provides a novel perspective for understanding disease mechanisms, but also offers valuable insights for the development of new therapies, the design of antioxidant strategies, and the exploration of fundamental biological questions. Furthermore, the successful development of this tool highlights its potential applications in precision medicine, providing technological support for personalized diagnosis and innovative drug development. In conclusion, this study advances the field of sulfenylation site prediction by integrating multidimensional features with deep learning techniques. It not only offers technical support for basic scientific research, but also opens new avenues for the future of precision medicine.

In future work, incorporating compression- and complexity-based measures of protein sequences may provide additional complementary features for improving predictive performance. Although in this study a Kolmogorov-complexity-inspired, compression-based evaluation was primarily used to assess feature sufficiency and to guide the resampling strategy, these measures could also be systematically explored as predictive features themselves. Integrating such complexity-aware representations with the proposed framework may further enhance its ability to capture subtle sequence-level information.

## Data Availability

The original contributions presented in the study are included in the article/Supplementary Material, further inquiries can be directed to the corresponding author. The source code implementing the proposed method is publicly available at https://github.com/zzhhhh666/BioSemAF-BiLSTM.
