# Supplementary material for: BioSemAF-BiLSTM: a protein sequence feature extraction framework based on semantic and evolutionary information
Source: Front Genet. 2025 Sep 17;16:1616880. doi: 10.3389/fgene.2025.1616880 (PMC12483914; doi:10.3389/fgene.2025.1616880)
Supplement: Supplementary file 1 [file Supplementaryfile1.pdf]

# Supplementary Material

## S1. ILLUSTRATION AND IMPLEMENTATION OF SMOTE AND SVMSMOTE

To address the pronounced class imbalance in our benchmark, we adopt two widely used oversampling strategies: *Synthetic Minority Over-sampling Technique (SMOTE)* (Chawla et al., 2002) and *Support Vector Machine Synthetic Minority Over-sampling Technique (SVMSMOTE)* (Demidova and Klyueva, 2017), implemented via the `imbalanced-learn` library https://imbalanced-learn.org. Both methods synthesize additional minority-class samples in *feature space* (e.g., fastText embeddings and PSSM vectors) rather than generating artificial amino acid sequences, thereby preserving the biochemical meaning of the raw sequence data.

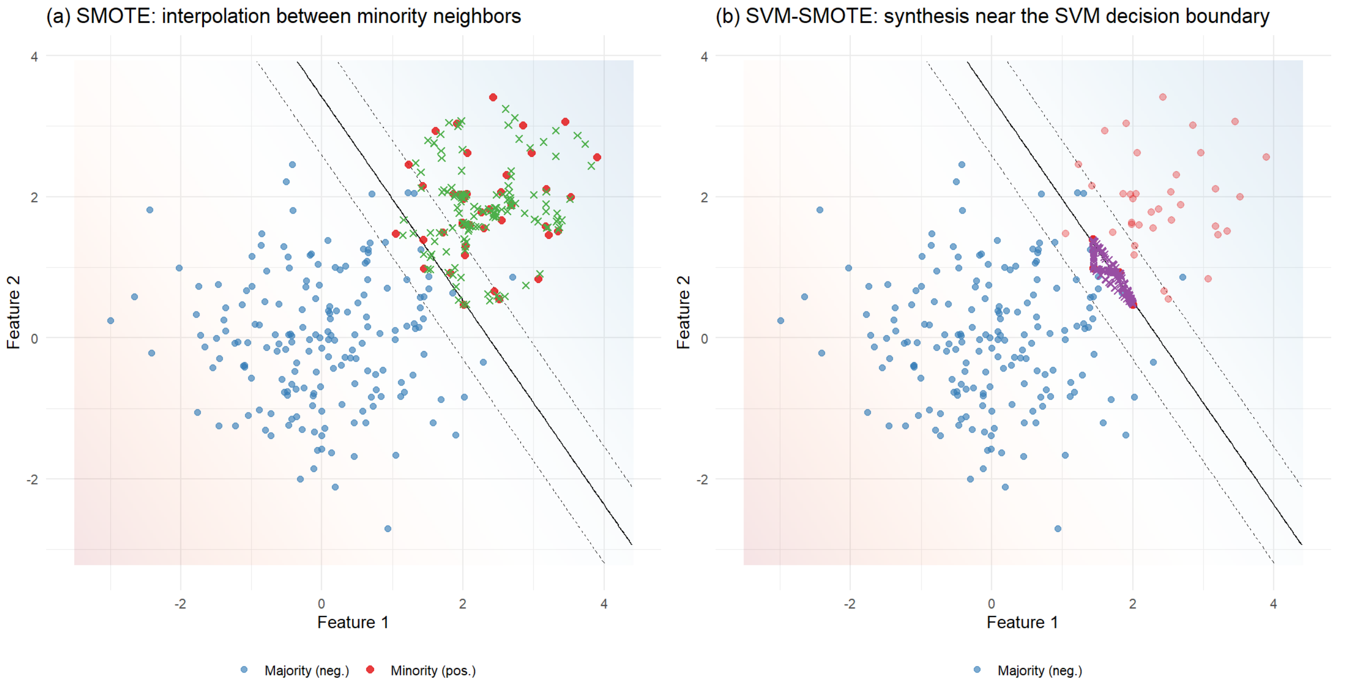


**Figure S1.** Schematic illustration of SMOTE and SVMSMOTE. **(a) SMOTE:** for each minority instance, a random neighbor among its $k$ nearest minority neighbors is selected, and a synthetic point is generated by linear interpolation along the segment connecting the two points in feature space. This tends to populate the minority region broadly but may produce redundant samples far from the decision boundary. **(b) SVMSMOTE:** a linear SVM is first trained to identify the decision boundary and margin. Synthetic samples are then generated *only* for minority instances near the boundary (support-vector or small-margin region), improving class separability while avoiding oversampling in safe interior regions. Solid line: SVM decision boundary; dashed lines: approximate $\pm 1$ margin. Blue: majority class; red: original minority samples; colored crosses: synthetic samples.

## Method Details

### SMOTE (Chawla et al., 2002).

Given a minority sample $\mathbf{x}$ and one of its $k$ nearest minority neighbors $\mathbf{x}_{\text{nbr}}$, SMOTE draws $\lambda \sim \mathcal{U}(0,1)$ and synthesizes

$$\mathbf{x}_{\text{new}} = \mathbf{x} + \lambda\,(\mathbf{x}_{\text{nbr}} - \mathbf{x}).$$

Repeating this procedure for randomly chosen bases and neighbors yields a user-specified number of synthetic minority samples, broadly enriching the minority region in feature space.

### SVMSMOTE (Demidova and Klyueva, 2017).

SVMSMOTE first trains a (typically linear) SVM on the imbalanced training set and computes the signed distances (margins) of minority samples to the decision boundary. Minority instances with small absolute margins (near the boundary) are selected as bases; synthetic samples are then generated via the same interpolation mechanism as SMOTE between those bases and their minority neighbors. This focuses synthesis where the classifier is most uncertain and tends to improve boundary modeling.

### Practical Notes.

(i) We oversample *only* in the learned feature space, not on raw symbol strings. (ii) We keep the original class distribution in the independent test set unchanged. (iii) Hyperparameters (*k* for neighbors, target sampling ratio, SVM kernel/penalty) are tuned on training splits; see main text for the chosen values.

## Example Implementation (imbalanced-learn)

Below is a minimal Python example showing how we apply SMOTE and SVMSMOTE with `imbalanced-learn`. Here, `X` are concatenated feature vectors (e.g., fastText embeddings + PSSM), and `y` are class labels.

```python
from imblearn.over_sampling import SMOTE, SVMSMOTE
from sklearn.svm import SVC

# X: feature matrix (fastText + PSSM), y: labels (0/1)
# 1) Plain SMOTE
smote = SMOTE(k_neighbors=5, sampling_strategy='auto', random_state=42)
X_sm, y_sm = smote.fit_resample(X, y)

# 2) SVMSMOTE (focus near SVM decision boundary)
svm_smote = SVMSMOTE(k_neighbors=5,
                     m_neighbors=10,
                     svm_estimator=SVC(kernel='linear', C=1.0),
                     sampling_strategy='auto',
                     random_state=42)
X_svm_sm, y_svm_sm = svm_smote.fit_resample(X, y)
```

## Why SVMSMOTE in Our Setting?

Because minority positives are scarce and often lie close to the decision boundary, SVMSMOTE concentrates synthesis in those critical regions, which empirically improves boundary modeling and yields

higher sensitivity while maintaining specificity. In contrast, plain SMOTE may generate redundant points in safe interior areas, offering less benefit for boundary refinement.

## Reproducibility

All experiments fix `random_state` seeds for resampling and model initialization. The independent test split from the benchmark remains untouched during resampling to avoid information leakage. See the code repository and main text for full training details.

# S2. PSEUDO CODE OF FEATURE INFORMATION LOSS COMPUTATION

## Symbolic Notation

| Symbol | Definition |
|---|---|
| $\mathcal{K}(X)$ | Kolmogorov complexity of raw sequence |
| $\mathcal{K}(f(X))$ | Kolmogorov complexity of feature representation |
| $\mathcal{K}(X, f(X))$ | Joint complexity of sequence and features |
| $\mathbb{B}$ | Byte stream space ($\mathbb{B} := \{0,1\}^*$) |
| $\mathcal{C}$ | Compression operator ($\mathcal{C} : \mathbb{B} \to \mathbb{B}$) |
| $\eta_{\text{loss}}$ | Information loss rate (ratio $\in [0, 1]$) |

## Pseudo Code

**Algorithm 1** Symbolic Information Loss Computation

**function** COMPUTEINFOLOSS($X, f(X)$)
  **Input:** ▷ $X \in \Sigma^*$ (amino acid sequence), $f(X) \in \mathbb{R}^d$
  **Output:** $\eta_{\text{loss}}$
  ▷ Compute $\mathcal{K}(X)$
  $B_X \leftarrow \text{Encode}(X)$ ▷ $B_X \in \mathbb{B}$
  $\hat{B}_X \leftarrow \mathcal{C}(B_X)$
  $\mathcal{K}(X) \leftarrow |\hat{B}_X|$
  ▷ Compute $\mathcal{K}(f(X))$
  $B_f \leftarrow \text{Vec2Bytes}(f(X))$ ▷ $B_f \in \mathbb{B}$
  $\hat{B}_f \leftarrow \mathcal{C}(B_f)$
  $\mathcal{K}(f(X)) \leftarrow |\hat{B}_f|$
  ▷ Compute $\mathcal{K}(X, f(X))$
  $B_{joint} \leftarrow B_X \oplus B_f$ ▷ $\oplus$: Concatenation
  $\hat{B}_{joint} \leftarrow \mathcal{C}(B_{joint})$
  $\mathcal{K}(X, f(X)) \leftarrow |\hat{B}_{joint}|$
  ▷ Compute $\eta_{\text{loss}}$
  $\eta_{\text{loss}} \leftarrow \frac{\mathcal{K}(X,f(X)) - \mathcal{K}(f(X))}{\mathcal{K}(X)}$
  **return** $\eta_{\text{loss}}$
**end function**

**procedure** MAIN
  $X \leftarrow \text{LoadSequence}(\Omega)$ ▷ $\Omega$: input space
  $f(X) \leftarrow \text{ExtractFeatures}(X)$
  $\eta_{\text{loss}} \leftarrow \text{ComputeInfoLoss}(X, f(X))$
  **Output** $\eta_{\text{loss}} \times 100\%$
**end procedure**

# REFERENCES

Chawla, N., Bowyer, K., Hall, L., and Kegelmeyer, W. (2002). Smote: Synthetic minority over-sampling technique. *J. Artif. Intell. Res. (JAIR)* 16, 321–357. doi:10.1613/jair.953

Demidova, L. and Klyueva, I. (2017). Svm classification: Optimization with the smote algorithm for the class imbalance problem. 1–4. doi:10.1109/MECO.2017.7977136
